# An Online Cross-Sectional Survey on Oral Healthcare Among School-Age Children During COVID-19 Epidemic in Wuhan, China

**DOI:** 10.3389/fmed.2021.572217

**Published:** 2021-06-22

**Authors:** Zhen Li, Yuhong Li, Chang Liu, Han Jiang, Chenzheng Zhang, Minquan Du

**Affiliations:** The State Key Laboratory Breeding Base of Basic Science of Stomatology (Hubei-MOST) & Key Laboratory of Oral Biomedicine Ministry of Education, School and Hospital of Stomatology, Wuhan University, Wuhan, China

**Keywords:** Wuhan, school-age children, oral health, epidemic, COVID-19

## Abstract

**Background:** Since the outbreak of Coronavirus disease 2019 (COVID-19), the government of China adopted many measures which changed people's lifestyle including oral health-related lifestyle to control the transmission. The aim of this study was to investigate oral health status, oral healthcare behaviors, and parental attitudes toward oral healthcare among school-age children in Wuhan during the COVID-19 outbreak and what the status would be when the outbreak is under control.

**Methods:** This study was an online cross-sectional survey facing elementary school students in Wuhan. The questionnaire was completed by children's parents or other family members. The information on demographic data, oral health status, oral healthcare behaviors, and parental attitudes toward oral healthcare was collected at the end of school closure. The chi-square test was used to test the association of different questionnaire items.

**Results:** A total of 18,383 subjects aged 6–13 years with complete data were included in this investigation, and 44.2% of them suffered pain or discomfort related to teeth and gums during the epidemic. While there might be an increasing need and concern of oral healthcare during the outbreak and even when the outbreak was controlled, the worry of infection made it difficult for people to meet their demands of dental attendance.

**Conclusion:** The risk of cross-infection during the treatment had a negative influence on parental attitudes toward dental attendance. Effective measures should be taken to meet people's demands of dental attendance.

## Introduction

Coronavirus disease 2019 (COVID-19) is a respiratory disease caused by a novel coronavirus named severe acute respiratory syndrome coronavirus 2 (SARS-CoV-2). The World Health Organization (WHO) officially recognized the outbreaks as a public health emergency of international concern on January 30, 2020 (1). As of June 8, 2020, a total of 6,931,000 confirmed cases and 400,857 deaths has been recognized in the whole world (2). This is a great challenge not only for China, but also for the world.

Oral disease is a major public health problem which affects children worldwide (3). Moreover, it would impact children's eating, speaking, emotional well-being, and general health when it happened to children (4). It is widely accepted that lifestyles are closely associated with oral disease (5), and great changes have happened on children's daily life since the outbreak of COVID-19.

Currently, the transmission of COVID-19 is regarded as starting with an animal-to-human transmission, followed by human-to-human spread. The modes of its interpersonal transmission include respiratory droplets, contact transmission, and aerosol routes. In addition, some asymptomatic spread cases were also reported (6). Considering these transmission characteristics of COVID-19, dentistry practices have a high risk of infection such as the frequent production of aerosols, constant presence of saliva, and the unavoidable touch of mouth, nose, and eyes (7). As a result, general non-emergency dental treatment was suspended and only emergency dental services were provided across China since January 2020 (8). Besides the limitation of dental attendance, many other measures, including lockdown for the whole Hubei province, quarantining infected and suspected people, restricting access to public spaces, and issuing a home quarantine order to all the residents, were adopted by the government of China to control the transmission efficiently. During this special domiciliary time, people have spent most of their time staying at home. For citizens in Wuhan, only online shopping provided by local supermarkets and markets was available and a narrow range of goods could be bought. This epidemic also has an indirect impact on people's lifestyle through psychology. It was reported that over half of Chinese suffered moderate-to-severe psychological impact during the initial phase of the COVID-19 outbreak (9).

With all of these changes, we hypothesized that the oral health status, the oral healthcare behaviors, and the attitudes toward oral healthcare of school-age children may have also changed. Wuhan, as the city facing the severest epidemic condition across China (10), may face more challenges than other cities on oral healthcare.

The aim of this study was to investigate oral health status, oral healthcare behaviors, and parental attitudes toward oral healthcare among school-age children in Wuhan during the COVID-19 outbreak and what the status would be when the outbreak is under control, thereby providing guidance for the following preventive and therapeutic oral healthcare in Wuhan and other districts which are affected by the epidemic.

## Materials and Methods

### Ethical Clearance

This questionnaire survey was undertaken by the Department of Preventive Dentistry, School & Hospital of Stomatology of Wuhan University. The questionnaire was anonymous to ensure privacy protection. Ethical approval (Approval no. HGGC-035) for the study was obtained from the Ethics Committee of the School & Hospital of Stomatology of Wuhan University, and informed consent was obtained from the guardian of each subject.

### Study Participants

This survey faces to elementary school students in Wuhan. In view of their age, parents or other family members are allowed to fill in the questionnaire instead.

Data collection was conducted from May 1 to 7, 2020. An online data collection was conducted because of the home quarantine order. The questionnaire was published on Wenjuanxing, and it was set as “per IP address and WeChat account can only submit the questionnaire once” through the platform to prevent double entry from participants. Then, we posted the link and the quick response code of the questionnaire, on WeChat groups and moments, attaching with a brief introduction, and asked people for passing on to their friends and relatives.

### Questionnaire Survey

The questionnaire consisted of four parts: (1) demographic data; (2) oral health status; (3) oral healthcare behaviors; and (4) attitudes toward oral healthcare during and after the epidemic situation.

Demographic data was collected on gender, age, and living region, including each district in Wuhan and the other regions in China.

To understand oral health status, we listed options including dental caries, bleeding while brushing teeth, loss of restorative material, tooth trauma, bad breath, oral ulcer, and other oral discomfort (an open-ended option) for people to multiply choose. In addition, we also afforded “I don't know” and “I don't have any discomfort” options. A self-assessment regarding oral health status was required in the end of the questionnaire.

In the aspect of oral healthcare behaviors, questions were designed covering several areas: (1) the choices about using oral healthcare products for the home; (2) the choices about using oral health services toward prevention; (3) meal times; (4) frequency of having confectionery; (5) frequency of having sweet drinks; (6) frequency of having sugared drinks like milk, yogurts, milk powders, tea, soybean milk, and coffees; and (7) frequency of toothbrushing.

Attitudes toward oral healthcare were measured by questions for during and after the epidemic situation, respectively, including the level of concern on oral health, the level of concern on access to treatment, the level of worry on being infected in the process of oral treatment, and the attitudes toward dental attendance.

In this questionnaire, “before the epidemic” means the period when there is no outbreak of epidemic (before January 23, 2020); “during the epidemic” means during Wuhan lockdown (from January 23, 2020, to April 8, 2020); and “after the epidemic” means the period that the epidemic is under control.

### Statistical Analysis

Data analysis was performed using the SPSS (PC version 23.0) software with the level of statistical significance set at *P* < 0.001. Subjects with missing data were excluded from the analysis.

The Optimal Binning method was used to select the optimal cutoff point of age. The chi-square test was used to test the association of different questionnaire items. McNemar's test was taken into account to find out the different attitudes toward oral healthcare in different epidemic situations.

## Results

A total of 18,694 subjects took part in this investigation. Of these subjects, 159 children were excluded because their living region was out of Wuhan and 152 subjects were excluded because of their age. At last, 18,383 subjects (9,753 boys and 8,630 girls) between the ages of 6 and 13 years were included in this study. The age range of 9–10 is the cutoff point between younger and older school-age children in this study. The characteristics of the study group are shown in Table 1.

**Table 1:** General characteristics of the study group.

	**Male**		**Female**		**Total**
	***N***	**%**	***N***	**%**	
**Age (years)**					
6	205	55.26%	166	44.74%	371
7	1,864	52.43%	1,691	47.57%	3,555
8	1,675	52.82%	1,496	47.18%	3,171
9	1,460	53.11%	1,289	46.89%	2,749
10	1,722	52.20%	1,577	47.80%	3,299
11	1,375	52.28%	1,255	47.72%	2,630
12	1,257	54.51%	1,049	45.49%	2,306
13	195	64.57%	107	35.43%	302
**Total**	9,753	53.05%	8,630	46.95%	18,383

Table 2 presents children's oral health status during the epidemic. Almost half of the children (44.2%) suffered oral discomfort, and the younger group had a higher proportion than the older group (47.5 vs. 40.3%). Besides, more children aged 6–9 years had an increase in the severity of oral discomfort than the children aged 10–13 years during the epidemic (7.6 vs. 4.7%). No significant difference was found for gender in the items above. As for detailed experience of pain/discomfort the children suffered, the younger group had a higher rate of dental caries and loss of restorative material than the older group while more girls than boys suffered bleeding while brushing teeth.

**Table 2:** Chi-square test for relationship of gender/age and oral health status during the epidemic.

	**Gender**					**Age**				***P*-value**	**Total**	
	**Male**		**Female**		***P*-value**	**6–9 years**		**10–13 years**				
	***N***	**%**	***N***	**%**		***N***	**%**	***N***	**%**		***N***	**%**
**Experience of pain/discomfort related to teeth and gums**					0.484					<0.001		
Yes	4,331	44.4%	3,788	43.9%		4,678	47.5%	3,441	40.3%		8,119	44.2%
No	5,422	55.6%	48,42	56.1%		5,168	52.5%	5,096	59.7%		10,264	55.8%
**Severity of oral pain/discomfort compared with that before the epidemic**					0.374					<0.001		
Increase	595	6.4%	505	6.1%		713	7.6%	387	4.7%		1,100	6.2%
No change	7,978	85.5%	7,130	86.2%		8,133	86.1%	6,975	85.4%		15,108	85.8%
Decrease	761	8.2%	636	7.7%		595	6.3%	802	9.8%		1,397	7.9%
**Detailed experience of pain/discomfort related to teeth and gums**												
Dental caries	1,511	35.8%	1,391	37.6%	0.246	1,888	41.1%	1,014	30.4%	<0.001	2,902	36.6%
Bad breath	1,173	27.8%	950	25.7%	0.031	1,109	24.2%	1,014	30.4%	0.194	2,123	26.8%
Oral ulcer	1,121	26.6%	916	24.7%	0.058	1,047	22.8%	990	29.7%	0.038	2,037	25.7%
Bleeding while brushing teeth	313	7.4%	397	10.7%	<0.001	358	7.8%	352	10.6%	0.087	710	9.0%
Loss of restorative material	256	6.1%	251	6.8%	0.241	351	7.6%	156	4.7%	<0.001	507	6.4%
Tooth trauma	35	0.8%	20	0.5%	0.115	29	0.6%	26	0.8%	0.901	55	0.7%

Table 3 provides information on oral healthcare behaviors. As for the use of oral healthcare products for the home, the proportion of subjects who used electric toothbrush (33.1 vs. 30.2%), fluoride toothpaste (26.1 vs. 20.2%), mouth rinse (16.1 vs. 7.0%), and dental floss (8.8 vs. 8.0%) after the epidemic was higher than that during the epidemic. What is more, little difference was found for gender and significant difference was found for age in the use of oral healthcare products for the home. No matter the situation was during the epidemic or after the epidemic, the younger group had a higher rate of the usage of electric toothbrush and fluoride toothpaste while the older group had a higher rate of the usage of mouth rinse.

**Table 3:** Chi-square test for relationship of age and oral healthcare behaviors during the epidemic.

	**6–9 years**		**10–13 years**		***P*-value**	**Total**	
	***N***	**%**	***N***	**%**		***N***	**%**
**Use of oral healthcare products for the home**							
**During the epidemic**							
Manual toothbrush	7,197	73.5%	6,349	75.0%	0.050	13,546	74.2%
Electric toothbrush	3,144	32.1%	2,361	27.9%	<0.001	5,505	30.2%
Fluoride toothpaste	2,085	21.3%	1,598	18.9%	<0.001	3,683	20.2%
Mouth rinse	532	5.4%	755	8.9%	<0.001	1,287	7.0%
Dental floss	799	8.2%	663	7.8%	0.383	1,462	8.0%
**After the epidemic**							
Manual toothbrush	5,768	60.0%	5,316	63.9%	<0.001	11,084	61.8%
Electric toothbrush	3,407	35.4%	2,527	30.4%	<0.001	5,934	33.1%
toothpaste	2,643	27.5%	2,040	24.5%	<0.001	4,683	26.1%
Mouth rinse	1,422	14.8%	1,463	17.6%	<0.001	2,885	16.1%
Dental floss	857	8.9%	715	8.6%	0.427	1,572	8.8%
**Use of oral health services toward prevention**							
**Before the epidemic**							
Routine check-up	2,912	29.6%	2,287	26.8%	<0.001	5,199	28.3%
Pit and fissure sealant	1,829	18.6%	1,792	21.0%	<0.001	3,621	19.7%
Fluoride varnish	1,226	12.5%	625	7.3%	<0.001	1,851	10.1%
Routine supragingival scaling	648	6.6%	954	11.2%	<0.001	1,602	8.7%
**After the epidemic**							
Routine check-up	5,668	57.6%	4,466	52.3%	<0.001	10,134	55.1%
Pit and fissure sealant	1,751	17.8%	1,009	11.8%	<0.001	2,760	15.0%
Fluoride varnish	1,281	13.0%	597	7.0%	<0.001	1,878	10.2%
Routine supragingival scaling	1,566	15.9%	1,855	21.7%	<0.001	3,421	18.6%

With regard to dental service utilization for preventive need, 28.3, 19.7, 10.1, and 8.7% of children took a routine check-up, pit and fissure sealant, fluoride varnish, and routine supragingival scaling before the epidemic, respectively. Moreover, the corresponding proportions were 55.1, 15.0, 10.2, and 18.6% when the epidemic was controlled. Children aged 6–9 years had a higher proportion of taking routine check-up (29.6 vs. 26.8%) and fluoride varnish (12.5 vs. 7.3%) than the older children and a lower proportion of taking pit and fissure sealant (18.6 vs. 21.0%) and routine supragingival scale (6.6 vs. 11.2%) before the epidemic. Besides, the parents of younger children had a higher willing of taking routine check-up (57.6 vs. 52.3%), fluoride varnish (13.0 vs. 7.0%), and pit and fissure sealant (17.8 vs. 11.8%) than the parents of older children and a lower willing of taking routine supragingival scale (15.9 vs. 21.7%) than the parents of older children after the epidemic. No dental service but routine check-up (26.4% for boys and 30.4% for girls before the epidemic; 54.0% for boys and 56.4% for girls after the epidemic) had a utilization difference for gender.

Table 4 shows the frequency of eating and toothbrushing. A percentage of 20.2% of children had a change of meal times. The proportion of children who had an increase in the frequency of confectionery, sweet drinks, and sugared drinks like milk, yogurts, milk powders, tea, soybean milk, and coffees was 18.4, 10.3, and 22.9%, respectively. With 7.4% of children who had an increase in the frequency of toothbrushing, only 62.3% of children brushed their teeth more than twice a day during the epidemic. The proportion of girls who had a stable meal time (81.3 vs. 78.4%) and who brush their teeth more than twice a day (65.3 vs. 59.7%) were higher than that of boys. The proportion of the younger group who had an increase in the frequency of meal times (18.2 vs. 16.7%) and who brush their teeth more than twice a day (64.2 vs. 60.2%) was higher than that of the older group.

**Table 4:** Chi-square test for relationship of gender/age and oral healthcare behaviors during the epidemic.

	**Gender**					**Age**					**Total**	
	**Male**		**Female**		***P*-value**	**6–9 years**		**10–13 years**		***P*-value**		
	***N***	**%**	***N***	**%**		***N***	**%**	***N***	**%**		***N***	**%**
**Meal times**					<0.001					<0.001		
Increase	1,832	18.9%	1,360	15.9%		1,779	18.2%	1,413	16.7%		3,192	17.5%
No change	7,598	78.4%	6,975	81.3%		7,788	79.6%	6,785	80.0%		14,573	79.8%
Decrease	262	2.7%	242	2.8%		222	2.3%	282	3.3%		504	2.8%
**Frequency increase of having desserts**												
Confectionery	1,736	17.9%	1,635	19.0%	0.053	2,007	20.5%	1,364	16.1%	<0.001	3,371	18.4%
Sweet drinks	1,055	10.9%	824	9.6%	0.004	1,062	10.8%	817	9.6%	0.007	1,879	10.3%
Sugared drinks like milk, yogurts, milk powders, tea, soybean milk, and coffees	2,289	23.6%	1,896	22.1%	0.014	2,355	24.0%	1,830	21.6%	<0.001	4,185	22.9%
**Frequency of toothbrushing**					<0.001					<0.001		
Twice a day or more	5,822	59.7%	5,635	65.3%		6,318	64.2%	5,139	60.2%		11,457	62.3%
Once a day	3,524	36.1%	2,759	32.0%		3,190	32.4%	3,093	36.2%		6,283	34.2%
Less often than daily	394	4.0%	223	2.6%		325	3.3%	292	3.4%		617	3.4%
Seldom or never	13	0.1%	13	0.2%		13	0.1%	13	0.2%		26	0.1%
**Frequency of toothbrushing**					<0.001					<0.001		
Increase	707	7.3%	650	7.5%		602	6.1%	755	8.9%		1,357	7.4%
No change	8,447	86.8%	7,591	88.1%		8,688	88.3%	7,350	86.3%		16,038	87.4%
Decrease	578	5.9%	377	4.4%		546	5.6%	409	4.8%		955	5.2%

Tables 5, 6 show parental attitudes toward oral healthcare during and after the epidemic situation. Table 5 shows a comparison of parental concern on oral health and access to oral treatment during the epidemic with that after the epidemic. A significant difference in the level of concern existed in different periods (*P*-value of McNemar's test <0.001). The levels of both concern on oral health and access to treatment after the epidemic were higher than those during the epidemic. Table 6 shows the relationship of worry on being infected in the process of oral treatment and the attitudes toward dental attendance after the epidemic. The overwhelming majority (93.8%) of the parents worried on being infected in the process of oral treatment after the epidemic, and half of the parents (53.4%) still avoided the dental attendance after the epidemic (no treatment for 9.8%, deal with it by oneself and avoid going to hospital for 13.4%, and avoid dental practice except for emergency cases for 30.2%).

**Table 5:** Parental attitudes toward the oral health care compared with that before the epidemic.

	**During the epidemic**		**After the epidemic**		***P*-value**
	***N***	**%**	***N***	**%**	**(McNemar's test)**
**The level of concern on oral health**					<0.001
Increase	5,484	30.1%	6,340	35.0%	
No change	12,161	66.7%	11,475	63.4%	
Decrease	589	3.2%	293	1.6%	
**The level of concern on access to treatment**					<0.001
Extreme	1,783	9.8%	21,66	12.1%	
Severe	2,340	12.9%	2,705	15.1%	
Moderate	1,546	8.5%	1,816	10.1%	
Mild	4,086	22.5%	3,547	19.8%	
Minimal	4,716	26.0%	4,263	23.7%	
Not at all	3,672	20.2%	3,455	19.2%	

**Table 6:** Chi-square test for relationship of the level of worry on being infected in the process of oral treatment and the attitudes toward dental attendance after the epidemic.

	**The attitudes toward dental attendance after the epidemic**									
	**No treatment**		**Deal with it by oneself and avoid going to hospital**		**Avoid dental practice except for emergency cases**		**Some of dental practice can be performed under adequate protection measures**		**Going to hospital for dental treatment after testing for COVID-9**		**All dental practice can be performed under adequate protection measures**		**All dental practice can be performed without adequate protection measures**		**Total**		***P*****-value**
	***N***	**%**	***N***	**%**	***N***	**%**	***N***	**%**	***N***	**%**	***N***	**%**	***N***	**%**	***N***	**%**	
**The level of worry on being infected in the process of oral therapy after the epidemic**																	<0.001
Extreme	843	16.3%	873	16.9%	1,534	29.7%	1,162	22.5%	476	9.2%	258	5.0%	11	0.2%	5,157	30.4%	
Severe	404	9.4%	625	14.6%	1,482	34.5%	1,281	29.8%	283	6.6%	209	4.9%	10	0.2%	4,294	25.3%	
Moderate	139	6.6%	254	12.0%	726	34.4%	720	34.1%	149	7.1%	115	5.4%	9	0.4%	2,112	12.5%	
Mild	157	5.0%	313	10.0%	923	29.5%	1,277	40.9%	264	8.5%	179	5.7%	11	0.4%	3,124	18.4%	
Minimal	48	4.0%	92	7.6%	247	20.5%	518	43.0%	128	10.6%	164	13.6%	9	0.7%	1,206	7.1%	
Not at all	72	6.9%	116	11.1%	202	19.3%	353	33.7%	130	12.4%	165	15.7%	10	1.0%	1,048	6.2%	
**Total**	1,663	9.8%	2,273	13.4%	5,114	30.2%	5,311	31.3%	1,430	8.4%	1,090	6.4%	60	0.4%	16,941	100.0%	

## Discussion

This study based on a large sample size with unequal gender distribution, which was resulted from the snowball sampling techniques and consisted with sex ratio imbalance in Wuhan (11).

The results of this study show that some changes happened on children's oral health status, oral healthcare behaviors, and parental attitudes toward oral healthcare in Wuhan during the COVID-19 outbreak. Besides, significant differences were found in oral healthcare between different age groups. What is more, while there was an increasing necessity and concern of oral healthcare because of the epidemic, the outbreak of the epidemic brought lots of difficulties for people to have a dental visit even when the epidemic was controlled.

### Oral Health Status

On the whole, the school-age children in Wuhan had a poor oral health and dental caries was the most common dental disease among this group. According to some recent reports on dental health among school-age children in China, the prevalence of dental caries was 46.88% for boys and 49.63% for girls among the children aged 6–12 years in Chengdu (12). In Shenzhen, the prevalence of dental caries was 55.7% for 6–9-year-old children and 31.9% for 10–13-year-old children (13). The prevalence of self-reported caries in this survey was 41.1% for children aged 6–9 years and 30.4% for children aged 10–13 years. It is relatively low compared with that in the previous study. A main explanation for this is that it is a result acquired in a self-reported way and it is difficult for people to distinguish whether it was caries or not.

Considering that studies on self-reported oral health and its correlation with clinical evaluation among school-age children were scarce, information about changes on self-reported oral health status during the epidemic was collected to identify what effect the epidemic had on oral health status.

Among the children who suffered pain/discomfort related to teeth and gums during the epidemic, the younger group had a higher proportion of subjects who had an increased severity than the older group. It indicated that the dental health status of the younger group was more susceptible to the epidemic than the older group. It could be partly explained by the fact that the younger group had a higher proportion of subjects who had a negative change on their diets during the epidemic than the older group.

What interested us is that a significant difference existed in the percentage of subjects who suffered discomfort related to teeth between different groups when the group was divided by age but not gender, while it was quite the opposite in the percentage of subjects who suffered discomfort related to gums. The former result that the younger group had a lower proportion of subjects who suffered discomfort related to teeth than the older group can be explained by the specific timing of tooth replacement (14). The latter result showing that the boys had a lower percentage of subjects who suffered discomfort related to gums than the girls was consistent with some of the previous studies (15), but it was difficult to explain combining the oral healthcare behaviors shown in this study. In this study, girls were more interested in adhering to the oral hygiene behaviors and their parents paid more attention to their children than the boys, which were reflected in the higher prevalence of routine check-up, recommended frequency of toothbrushing, and stable meal times. All of these results indicated that the girls might have a better oral hygiene level than boys. This difference in behaviors was consistent with previous studies. Furthermore, in most of such studies, boys had a worse gum health status (16–19). The result in this study was probably caused by physiological character difference in gender, such as the specific timing of teeth replacement and the fluctuation of hormone levels with stress events (20). Further researches are needed on it.

### Oral Healthcare Behaviors

The willingness to use oral healthcare products for the home and oral health services toward prevention had an increase when the epidemic was controlled. Generally, the younger group had a higher proportion of using recommended oral healthcare products and oral health services than the older group during and after the epidemic. It might be because parents tend to put more concern on the younger children.

### Parental Attitudes Toward Oral Healthcare

The changes on oral health status and oral healthcare behaviors indicated that there is an increase in the demand for oral healthcare after the epidemic. Besides, a large proportion of parents had an increased level of concern on oral health during and after the epidemic according to the results from parental attitudes toward oral healthcare.

However, 53.4% of them still have a tendency to avoid going to the hospital and the proportion of people who had concern on access to treatment after the epidemic was 80.8%. It can be partly explained by the positive correlation between the level of worry on being infected in the process of oral treatment and the attitudes toward dental attendance considering the large proportion of people who had worry on being infected in the process of oral treatment after the epidemic (93.8%).

Taking all these factors into account, more attention should be paid to spread information about the measures that have been taken to reduce the risk of cross-infection during the treatment.

### Strengths and Limitations

This study has both strengths and limitations. A major strength of the current study is that it is the first study to report the oral healthcare of school-age children in Wuhan during the COVID-19 outbreak. Wuhan has its specificity for it was the most serious district in China and has been under the strictest management for the longest time. Besides, it has a large sample size and the data was obtained before the epidemic passed.

There are also some limitations. Firstly, selection bias exists because of the snowballing sampling strategy and the use of an online survey. Secondly, recall bias exists as the oral health condition was reported by the parents. Thirdly, to attract more subjects to fill the questionnaire, the number of the questions was limited. As a result, many items were not able to have a further discussion.

## Conclusions

In conclusion, children's oral health status was affected by the epidemic, especially 6–9-year-old children. More attention should be paid on the eating habits of children aged 6–9 and toothbrushing habits of boys. Parents in Wuhan had more concerns on oral healthcare during and after the epidemic than before the epidemic state. Besides, the findings from this study show a high amount of dental treatment needed among school-age children in Wuhan after the epidemic. However, the worry on being infected in the process of oral treatment after the epidemic might be a major factor which would stop people from dental attendance. Effective measures should be taken to meet people's demands of oral healthcare. What is more, age-specific strategies in prevention are necessary for school-age children.

## Data Availability Statement

The raw data supporting the conclusions of this article will be made available by the authors, without undue reservation.

## Ethics Statement

The studies involving human participants were reviewed and approved by Ethics Committee of the School & Hospital of Stomatology of Wuhan University. Written informed consent to participate in this study was provided by the participants' legal guardian/next of kin.

## Author Contributions

All authors listed have made a substantial, direct and intellectual contribution to the work, and approved it for publication.

## Conflict of Interest

The authors declare that the research was conducted in the absence of any commercial or financial relationships that could be construed as a potential conflict of interest.
